# Non-targeted metabolomics analysis of metabolite changes in two quinoa genotypes under drought stress

**DOI:** 10.1186/s12870-023-04467-6

**Published:** 2023-10-20

**Authors:** Xiaolin Zhu, Mingjun Zhang, Baoqiang Wang, Xinrong Song, Xian Wang, Xiaohong Wei

**Affiliations:** 1 College of Agronomy, Gansu Agricultural University, Lanzhou, 730070 China; 2 Gansu Provincial Key Laboratory of Aridland Crop Science, Gansu Agricultural University, Lanzhou, 730070 China; 3 College of Plant Protection, Gansu Agricultural University, Lanzhou, 730070 China; 4 College of Life Science and Technology, Gansu Agricultural University, Lanzhou, 730070 China

**Keywords:** Quinoa, Drought stress, Physiological characteristics, Metabolome

## Abstract

**Background:**

Quinoa is an important economic crop, drought is one of the key factors affecting quinoa yield. Clarifying the adaptation strategy of quinoa to drought is conducive to cultivating drought-tolerant varieties. At present, the study of quinoa on drought stress-related metabolism and the identification of related metabolites are still unknown. As a direct feature of biochemical functions, metabolites can reveal the biochemical pathways involved in drought response.

**Result:**

Here, we studied the physiological and metabolic responses of drought-tolerant genotype L1 and sensitive genotype HZ1. Under drought conditions, L1 had higher osmotic adjustment ability and stronger root activity than HZ1, and the relative water content of L1 was also higher than that of HZ1. In addition, the barrier-to- sea ratio of L1 is significantly higher than that of HZ1. Using untargeted metabolic analysis, a total of 523, 406, 301 and 272 differential metabolites were identified in L1 and HZ1 on day 3 and day 9 of drought stress. The key metabolites (amino acids, nucleotides, peptides, organic acids, lipids and carbohydrates) accumulated differently in quinoa leaves. and HZ1 had the most DEMs in Glycerophospholipid metabolism (ko00564) and ABC transporters (ko02010) pathways.

**Conclusion:**

These results provide a reference for characterizing the response mechanism of quinoa to drought and improving the drought tolerance of quinoa.

**Supplementary Information:**

The online version contains supplementary material available at 10.1186/s12870-023-04467-6.

## Introduction

Drought is extremely unfavorable to agriculture and livestock, especially drought is one of the key environmental stress factors affecting crop growth and production [1]. Drought stress in plants is physiologically complex and includes osmotic stress and specific ion toxicity. Drought stress in plants is associated with nutritional imbalances, reduced cell division and expansion, and excessive production of reactive oxygen species (ROS) [2]. The toxicity of excessively produced ROS triggers a cascade of oxidative reactions, resulting in enzyme inactivation and increased lipid peroxidation, the final product of which is malondialdehyde (MDA). When exposed to water stress conditions, many plants enhance the activity of antioxidant enzymes associated with increased proline concentration [3]. Proline plays an important role in osmotic pressure regulation, allowing cells to retain more water. In addition, the amino acid also shows plant defense characteristics, as a ROS scavenger and as a regulator of cellular redox status [4]. Therefore, the accumulation of proline in plants is considered be a positive indicator of plant tolerance to water stress. The ability of plants to retain water during the drying process is an important strategy for plant tolerance to stress caused by water stress. Therefore, the evaluation of relative water content changes is the best representative and rapid method to evaluate the genetic differences between water deficit and physiological water status of plants after water stress treatment. In addition, drought stress led to the accumulation of osmotic regulators (soluble sugar (SSC) and soluble protein (SP)) in cells to maintain cell swelling and stress. At the same time, leaves are the main organs for photosynthesis and respiration of plants, and their internal anatomical structures are also different under drought stress. Therefore, the study of leaf anatomical characteristics is helpful to reveal the effects of drought stress on plants [5]. For example, water shortage may lead to cell membrane rupture, cell wall thickening and mechanical tissue developed, vacuoles increased inorganic salt content ; the leaves are few and small, the cuticle is thickened, the mechanical tissue is developed, the stomata are many but small, and the ratio of palisade tissue / spongy tissue is reduced [5, 6]. At the same time, stomata are physiological organ structures closely related to the function of leaves, which are closely related to drought resistance. Plants can use stomatal regulation to cope with drought stress during drought [6]. Under drought stress, the thickness of spongy tissue and lower epidermis of *Eucommia ulmoides* leaves decreased significantly, and the stomatal area, stomatal size and stomatal aperture decreased [7]. Li ‘s research showed that drought stress reduced leaf epidermis, spongy tissue, leaf vein diameter and spongy ratio, stomatal aperture [7]. Thus, plants through the photosynthetic system, antioxidant system and respiratory system and other ways to deal with drought stress on plant damage.

At present, there are about 200,000-1000000 metabolites in plants [8]. Under stress, plants produce hundreds of metabolites, and the changes in the types and quantities of metabolites can reflect the adaptability of plants to the environment [9]. Plant metabolites play an important role in growth, cell integrity, energy storage, cell signal transduction, plant resource allocation, plant development and stress response [10]. When plants face drought stress, they usually respond by leaf rolling, stomatal closure, growth and development inhibition [11]. At the same time, some metabolites closely related to these reactions change significantly [12]. Plants change their physiological functions to adapt to different conditions through the changes of metabolites [13]. Studies have shown that the content of organic acids, sugars, sugar alcohols, amino acids and some soluble secondary metabolites changed significantly under drought stress [14]. For example, in the study of chickpea, it was found that the accumulation levels of metabolites such as choline, phenylalanine, alanine, tyrosine, glucosamine, guanine and aspartic acid decreased under drought stress [15]. The level of branched-chain amino acids in drought-tolerant barley increased under drought conditions [16]. 4-hydroxycinnamic acid and ferulic acid in rice are considered to be key metabolites of drought tolerance [17]. In addition, six metabolites: 3-cyanoalanine, phenylalanine, quinic acid, asparagine, p-benzoquinone and phytosphingosine were identified as potential biological markers of cherry drought response [18].

Quinoa (*Chenopodium quinoa* Willd) is an annual dicotyledonous herb. Its seeds contain all the nutrients required by the human body, and the proportion of amino acids is balanced. It has extremely high edible value and medical and health care value such as anti-cancer [19]. It has the biological characteristics of cold tolerance, drought tolerance, salt tolerance and barren tolerance. However, long-term drought stress will also seriously affect its yield and quality. Metabolomics is a powerful tool that can be used to fully understand the regulation of metabolic networks. It analyzes the mechanism of plants responding to changes in the external environment by studying the collection of all metabolites in cells at a certain time. Among them, untargeted metabolomics is committed to detecting, identifying, and quantifying as many metabolites as possible in a single or integrated analysis without the need for a priori knowledge of reliable criteria or annotated metabolites [20], the identification of metabolite signatures in non-targeted metabolomics relies heavily on searching existing databases for MS/MS or MSN profiles, such as MassBank [21], Metlin [22], and the global network of natural products social molecules (GNPS) [23]. Therefore, it is widely used to elucidate the molecular mechanisms of plant response and defense to various stresses [12]. The flowering period of quinoa was identified as the most sensitive stage to drought. The mechanisms of drought resistance in quinoa have been explored [24–27]. Abscisic acid (ABA) concentrations in the roots of Plateau Quinoa ‘INIA-Illpa’ and leaves of ‘Titicaca’ are increased under drought conditions, resulting in reduced stomatal guard cell inflation [27]. Furthermore, during ‘Titicaca’ drought stress, the concentration of ABA in the xylem increased faster in the branches than in the roots [27]. The synthesis of reactive oxygen species (Ros) scavengers is also an important aspect of quinoa response to drought stress, such as the accumulation of soluble sugars and proline [25]. Under drought stress, quinoa seedlings adapt to drought conditions by increasing osmotic regulator content, enhancing antioxidant enzyme activity, and scavenging reactive oxygen species in the body. Quinoa can also trigger high water use efficiency by reducing water loss through stomatal closure, regulation of cellular water deficit, and formation of a responsive mechanism for rhizome-to-stem ratios [26, 27]. At present, there are few studies on the metabolic regulation of quinoa drought tolerance. Drought-related physiological and metabolic changes may help to determine the sensitivity or tolerance of plants under water deficit conditions and can be used as stress indicators. Here, polyethylene glycol (PEG) 6000 was used to simulate the leaves of quinoa seedlings under drought stress. The physiological characteristics of quinoa (sensitive and tolerant) after drought treatment were first examined, and the changes in metabolites were comprehensively analyzed by ultra-high performance liquid chromatography and tandem mass spectrometry (UPLC-MS/MS) targeting techniques. The results of this study will help us better understand the metabolic changes of quinoa leaves in response to drought stress, determine the possible metabolomics characteristics of quinoa and the physiological adaptation mechanism of quinoa tolerance to drought stress, and provide a theoretical basis for quinoa drought resistance breeding. These results provide insights into the metabolites involved in the mechanisms of drought tolerance in plants, which will ultimately contribute to future genetic and metabolomic studies of domesticated crops. To our knowledge, this is the first metabolic comparison of quinoa leaf samples by GC-MS and LC-MS analysis.

## Methods

### Plant materials and treatments

This experiment was carried out under greenhouse conditions in College of Life Science and Technology of Gansu Agricultural University. In this study, drought-sensitive genotype HZ and drought-tolerant genotype Longli No.1 (L1 ) were selected for research, both from Gansu Academy of Agricultural Sciences, they were identified by Gansu Academy of Agricultural Sciences. Pest-free, plump and size - consistent quinoa seeds were selected for this study. Quinoa seeds were washed three times with distilled water, soaked in 95% ethanol for 5–6 min, and finally disinfected with 1% sodium hypochlorite for 5 min. Seeds were sown in pots containing 2 kg sandy loam soil, 10 seeds per pot, covered with 0.1 cm thick vermiculite, placed in a greenhouse, and watered normally. During the experiment, the greenhouse environment was maintained at 25 °C / 18 ± 1 °C (day/night), and the relative humidity was 75 ± 5%. When quinoa seedlings grew to 2 months(Four leaves one heart period), the experimental group was treated with 20% polyethylene glycol-6000 (PEG-6000) to simulate osmotic stress (ψs = -0.49 MPA), and the control group was well watered. All quinoa leaves were taken on days 0,3,6,9 and 12 after treatment. Each biological replicate contained six individual plants under drought stress. The sample leaves were taken from top to bottom from the third to fourth leaves.

### Sample preparation

Leaves were collected from control and drought plants at noon on 3 and 9 days of drought stress. Immediately after collection, freeze in liquid nitrogen and store at -80 ° C. Samples were lyophilized for 72 h and ground using TissueLyser, and lyophilized powders (50 mg) were used for untargeted global metabolite analysis based on UPLC-HRMS. 50 mg samples were weighed, and 1000µL of extract containing internal standard ( 1000:2 ) ( methanol acetonitrile water volume ratio = 2:2:1, internal standard concentration 2 mg/L) was added, and vortexed for 30 s ; add ceramic beads, 45 Hz grinder processing 10 min, ultrasonic 10 min ( ice water bath ) ; stand at -20 °C for one hour ; the sample was centrifuged at 4 °C, 12,000 rpm for 15 min; carefully remove 500 µL supernatant in EP tube ; drying the extract in a vacuum concentrator ; the dried metabolites were redissolved with 160µL extract ( acetonitrile-water volume ratio:1: 1). Vortex 30 s, ice bath ultrasound 10 min; the sample was centrifuged at 4 °C, 12,000 rpm for 15 min; carefully take out 120µL supernatant in 2mL injection bottle, each sample take 10µL mixed into QC samples for testing.

### UPLC-HRMS analysis

Undetermined metabolomics analysis was performed on UPLC-HRMS (model: Acquity I-Class PLUS and Xevo G2-XS Q Tof mass spectrometer). Chromatographic separation was obtained on an Acquity UPLC HSS T3 column (1.8 μm 2.1*100mm). Both positive and negative ion modes were: mobile phase A: 0.1% formic acid aqueous solution; mobile phase B: 0.1% formic acid acetonitrile, injection volume was 1uL, the total running time of each sample was 51.35 min. Waters Xevo G2-XS QTOF high resolution mass spectrometer can collect primary and secondary mass spectrometry data in MSe mode under the control of the acquisition software (MassLynx V4.2, Waters). In each data acquisition cycle, dual-channel data acquisition can be performed on both low collision energy and high collision energy at the same time. The low collision energy is 2 V, the high collision energy range is 10 ~ 40 V, and the scanning frequency is 0.2 s for a mass spectrum. The parameters of the ESI ion source are as follows: capillary voltage: 2000 V (positive ion mode) or-1500 V (negative ion mode); cone hole voltage: 30 V; ion source temperature: 150 °C; desolvation gas temperature 500 °C; reverse blowing gas flow rate: 50 L / h; desolvation gas flow rate: 800 L/h. mass nucleus ratio (m/z) Collection range: 50-1200.

### Data processing

The raw data collected using MassLynx V4.2 is processed by Progenesis QI software for peak extraction, peak alignment and other data processing operations, based on the Progenesis QI software online METLIN database and Biomark’s self-built library for identification, and at the same time, theoretical fragment identification and mass deviation All are within 100ppm.

Principal Component Analysis (PCA) was performed with R language (R-3.1.1) and software package: scales, GGPLOT2, GGREPEL, scatterplot3d. It was used for preliminary understanding of the overall metabolic differences between groups and the magnitude of variability between groups. R language (R-3.1.1) was used for correlation analysis and software package: pheatmap. The difference multiple was analyzed by R language (R-3.1.1), software package: GGPLOT2. Orthogonal projections tolatent structures- discriminant analysis (OPLS-DA) was used to maximize differences in metabolic characteristics between control and drought groups, enabling detection of metabolites present in biological samples, using r language (R-3.1.1), software Package: ropls [28], VIP threshold calculated by OPLS-DA model: ≥1; difference multiple threshold: ≥2 or ≤ 1/2.

### Physical characteristics

#### Determination of physiological parameters

Soluble protein was determined by Coomassie brilliant blue method [29]. Soluble sugar content was determined according to Wei ' s method [30]. The proline content was determined according to Bates ' method [31]. MDA, root activity and relative water content were determined according to Dhindsa method [32].

### Observation of Leaf Cell microstructure

One leaf of quinoa was selected from each replicate and repeated for 3 times. A 1.5 cm × 1.5 cm cube was cut from the middle of the leaf connected to the midrib, fixed in FAA fixative (70% ethanol 90 mL + formaldehyde 5 mL + acetic acid 5 mL), and stored in a refrigerator at 4 °C for routine paraffin sectioning. The materials were fixed by FAA for more than 24 h, dehydrated and transparentized by ethanol and xylene series, immersed in wax, and embedded. Slices (slice thickness 8 μm) were stained with safranine-fast green, and then sealed with neutral gum to make permanent sections. The sections were observed and photographed under a microscope. Each treatment was observed in 6 fields of vision. The structural parameters of leaf palisade tissue, sponge tissue, upper epidermis, lower epidermis and veins were observed and analyzed by ImageJ software.

#### Scanning electron microscope observation of stomatal structure of leaf lower epidermis

One quinoa leaf was selected from each replicate and repeated three times. A 1 cm × 1 cm square was cut from the side of the main vein in the middle of the leaf, fixed in an electron microscope fixative (2.5% glutaraldehyde), and stored in a refrigerator at 4 °C for electron microscope scanning. Each treatment was observed under a scanning electron microscope and photographed in 15 fields. The number of stomata and the number of closed stomata were counted from the field of view of a 100 × mirror, and the stomatal density (number/mm2) and stomatal closure percentage were calculated. The area, length and width of each stoma were measured by ImageJ software in two 400 × visual fields. The length of stoma was the longest value parallel to stoma and the width of stoma was the widest value perpendicular to stoma. The average values of area, length and width of stoma in two visual fields were calculated. Each treatment was repeated 3 times and the results were averaged.

1. Stomatal density (number·mm-2) = stomatal number / visual field area.
2. Percentage of closed stomata (%) = Number of closed stomata per visual field / Number of stomata per visual field × 100.

### Statistical analysis

SPSS 13.0 was used to analyze the physiological parameters. All treatments were repeated 3 times. Mean values and calculated standard errors are reported.

## Results

### Effects of PEG stress on osmotic adjustment substances and MDA in quinoa leaves

**Fig. 1:**
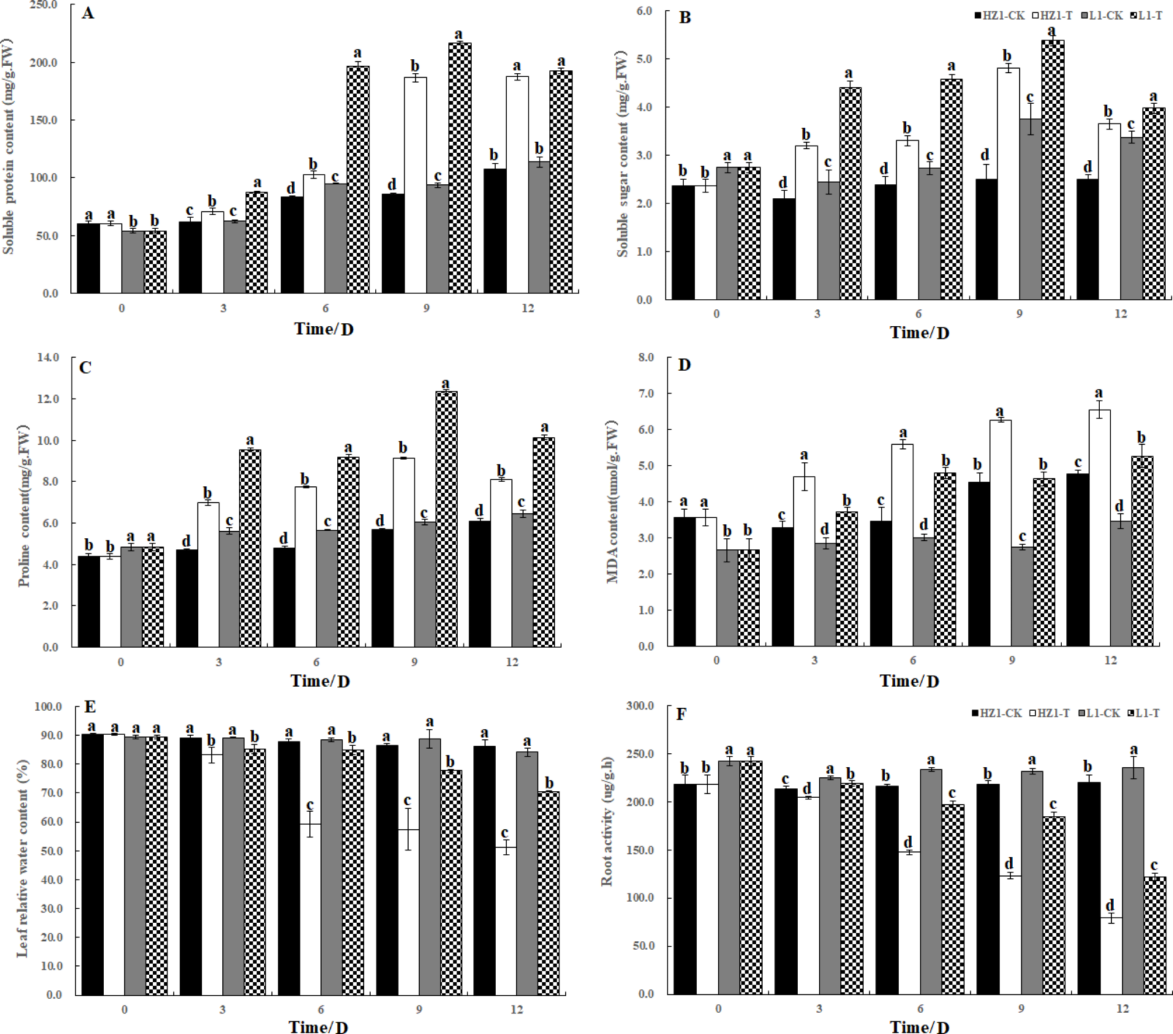
Effects of drought stress on osmotic adjustment substances, root activity and relative water content in leaves of Quinoa

As shown in Fig. 1A-F, the cent of soluble protein and MDA increased continuously with the prolongation of drought time in HZ1. The content of soluble protein increased by 118.05% compared with the control at 9 days after stress, and the content of MDA increased by 38.15% compared with the control. The content of soluble sugar and proline increased first and then decreased after PEG stress. The content of soluble sugar increased by 91.74% compared with the control at 9 days, and the content of proline increased by 60.84% compared with the control. In L1, with the continuous drought time, the contents of soluble protein, soluble sugar and proline increased first and then decreased, but they were higher than those of the control. The content of soluble protein increased by 131.05% compared with the control at 9 days after stress, and the content of soluble sugar increased by 43.40% compared with the control at 9 days after stress. The content of MDA increased continuously during the stress period, and increased by 68.92% compared with the control at 9 days after stress. In addition, with the duration of drought stress, the relative water content of quinoa seedling leaves showed a decreasing pattern. The RWC in HZ1 decreased by 6.73%, 32.55%, 33.63% and 40.62% at 3,6,9 and 12 days after stress, respectively. The RWC in L1 decreased by 4.44%, 3.99%, 12.31% and 16.16%, respectively. In addition, this study found that with the prolongation of drought stress days, the root activity of quinoa decreased continuously in both materials. On the third day of drought stress, the root activity of the two materials began to decrease significantly, and reached the minimum on the 12 th day after stress. The comparison between materials showed that the root activity of L1 was always higher than that of HZ1 after stress, and was 7.17% (day 3), 33.46% (day 6), 49.96% (day 9) and 54.23% (day 12) higher than that of HZ1, respectively.

### Effects of Drought stress on Leaf Anatomical structure of Quinoa

Combined with early physiological indicators, the 9th day quinoa leaves were selected to observe the effects of drought stress on leaf anatomical structure (Table 1). It can be seen from Figure S1 that the anatomical structure of the leaves in the control group was filled with cells, the structure was clear, the intercellular space was small, and the arrangement was neat. The thickness of the upper and lower epidermis of L1-CK was 14.38 μm and 12.51 μm, and the thickness of the upper and lower epidermis of L1-T was 14.54 μm and 9.52 μm. The thickness of the lower epidermis of L1-T was significantly lower than that of L1-CK, and decreased by 23.86% compared with L1-CK. The thickness of palisade tissue of L1 increased by 52.43% compared with the control after drought stress. In addition, the ratio of grid to sea under drought stress of L1 was significantly higher than that of the control, with a value of 1.11. The thickness of sponge tissue under drought stress in HZ1 was significantly higher than that of the control, and increased by 16.07% compared with the control, and the ratio of palisade tissue to spongy tissue under drought stress was lower than that of the control.

**Table 1:** Leaf mesophyll structure parameters of quinoa under drought stress

	Leaf mesophyll structure parameters				
Treatment	Upper epidermis thickness (µm)	Lower epidermis thickness (µm)	Palisade tissue thickness (µm)	Spongy tissue thickness (µm)	Ratio of palisade tissue and spongy tissue
L1-CK	14.38 ± 0.13Bb	12.51 ± 0.16Bb	39.45 ± 2.78Cc	54.25 ± 6.19Cc	0.73 ± 0.06Bb
L1-T	14.54 ± 0.04Bb	9.52 ± 0.13Cc	60.13 ± 6.07Aa	56.11 ± 9.78Cc	1.11 ± 0.26Aa
HZ1-CK	15.42 ± 0.19Aa	13.39 ± 0.11Aa	57.38 ± 3.75Bb	72.69 ± 5.16Bb	0.79 ± 0.08Bb
HZ1-T	15.11 ± 0.06Aa	13.66 ± 0.09Aa	57.62 ± 3.68Bb	84.37 ± 12.91Aa	0.70 ± 0.12Bb

### Effects of drought stress on stomatal characteristics of quinoa leaf lower epidermis

Figure S2 shows the stomatal distribution and stomatal characteristics of the lower epidermis of quinoa leaves under drought stress. We found that the stomatal length, stomatal width, stomatal area, stomatal density and stomatal opening number per unit area of the two materials under drought stress were lower than those of the control (Table 2). The stomatal length, stomatal width, stomatal area and stomatal density in L1 decreased by 39.78%, 52.54%, 48.22% and 12.22% respectively compared with the control, while the number of stomatal opening per unit area increased by 50% compared with the control. Stomatal length, stomatal width, stomatal area, stomatal density and stomatal opening number per unit area in HZ1 decreased by 31.42%, 34.44%, 61.60%, 15.56% and 15.39% respectively compared with the control.

**Table 2:** Leaf stomatal structure eigenvalue of lower epidermis of quinoa under drought stress

	Leaf stomatal structure eigenvalue of lower epidermis				
Treatment	Length of stomatal aperture(µm)	Width of stomatal aperture(µm)	Stomatal area (µm 2 )	Stomatal aperture (No.·mm^− 2^ )	Number of stomatal openings per unit area
L1-CK	8.97 ± 0.92Bb	2.05 ± 0.26Bb	10.91 ± 0.36Bb	45 ± 1.41Aa	7 ± 1.41Bb
L1-T	5.99 ± 0.05Dd	1.32 ± 0.36Dd	5.60 ± 0.12Dd	39.5 ± 0.71Bb	10.5 ± 0.71Aa
HZ1-CK	10.80 ± 0.83Aa	2.79 ± 0.14Aa	22.47 ± 2.89Aa	45 ± 4.24Aa	6.5 ± 0.71BCb
HZ1-T	7.41 ± 0.76Cc	1.83 ± 0.09Cc	8.63 ± 0.59Cc	38 ± 1.41Bb	5.5 ± 0.71Cc

### Metabolite sample quality control analysis

**Fig. 2:**
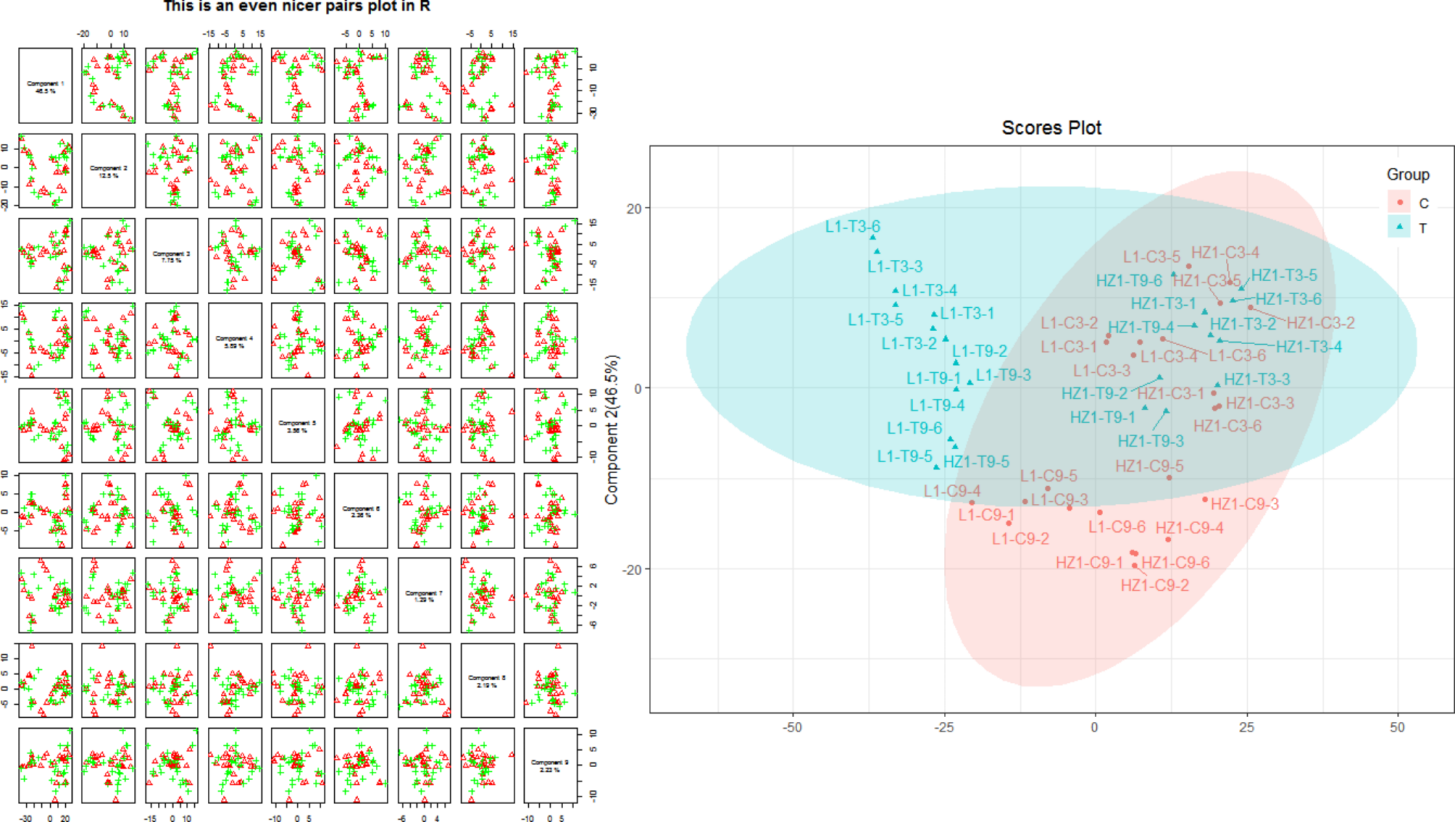
Partial least square discriminant analysis and 2D scores loading plot for the quinoa HZ1 and L1 under control and drought treatments at two time points (3 and 9 days). Samples at control and drought treatments did not overlap with each other, indicating an altered state of metabolite levels in the quinoa leaves. HZ1, sensitive variety; L1, tolerant variety

PLS-DA analysis was performed on drought and control conditions of drought-tolerant and drought-sensitive genotype materials at two different time points (Fig. 2). The first PLS component (PC1) explained 46.5% of the total variation, while the second component (PC2) explained 12.5% of the variation for the entire dataset. The fractional plot between PC1 and PC2 shows two different groups associated with drought and control samples. It shows that there are obvious differences in metabolite accumulation under two conditions. The sensitive genotype material (HZ1) and the tolerant genotype material (L1) samples were separated from each other under drought and control conditions, especially under drought conditions.

### Metabolite KEGG annotation

The KEGG annotation analysis showed that 846 metabolites (Figure S3) were involved in 13 primary pathways, including Global and overview maps (804), Biosynthesis of other secondary metabolites (615), Metabolism of terpenoids and polyketides (152), Amino acid Metabolism, metabolism of cofactors and vitamins (94), Lipid Metabolism (79), Carbohydrate Metabolism (29), Membrane transport (21), Glycan biosynthesis and Metabolism (20), Metabolism of other amino acids (18), Energy Metabolism (10), Translation (8) and Nucleotide Metabolism (7). Secondly, these 13 primary pathways contain a total of 153 secondary pathways, the 13 primary pathways contain 14,30,10,8,9,20,12,3,11,9,21,2 and 4 secondary pathways respectively. Furthermore, 113 of 846 metabolites were found to be annotated into 64 pathways by analysis, with the Metabolic pathways (KO01100) having the largest number of metabolites (75) ; They are Biosynthesis of metabolites secondary (KO01110) pathway (46), ABC transporters (KO02010) pathway (7), Phenylalanine metabolism (Ko00360) pathway (7), Tropane, piperidine and pyridine alkaloid Biosynthesis (KO00960) pathway (7), Tryptophan metabolism (KO00380) pathway (6), alpha-Linolenic acid metabolism (KO00592) pathway (5), Biosynthesis of amino acids (KO01230) pathway (5), Glucosinolate Biosynthesis (KO00966) pathway (5), Phenylpropanoid Biosynthesis (KO00940) pathway (5), the other 54 metabolic pathways had less than 5 metabolites.

### PCA and OPLS-DA were used to analyze the changes of metabolites in leaves of different quinoa cultivars under drought stress

**Fig. 3:**
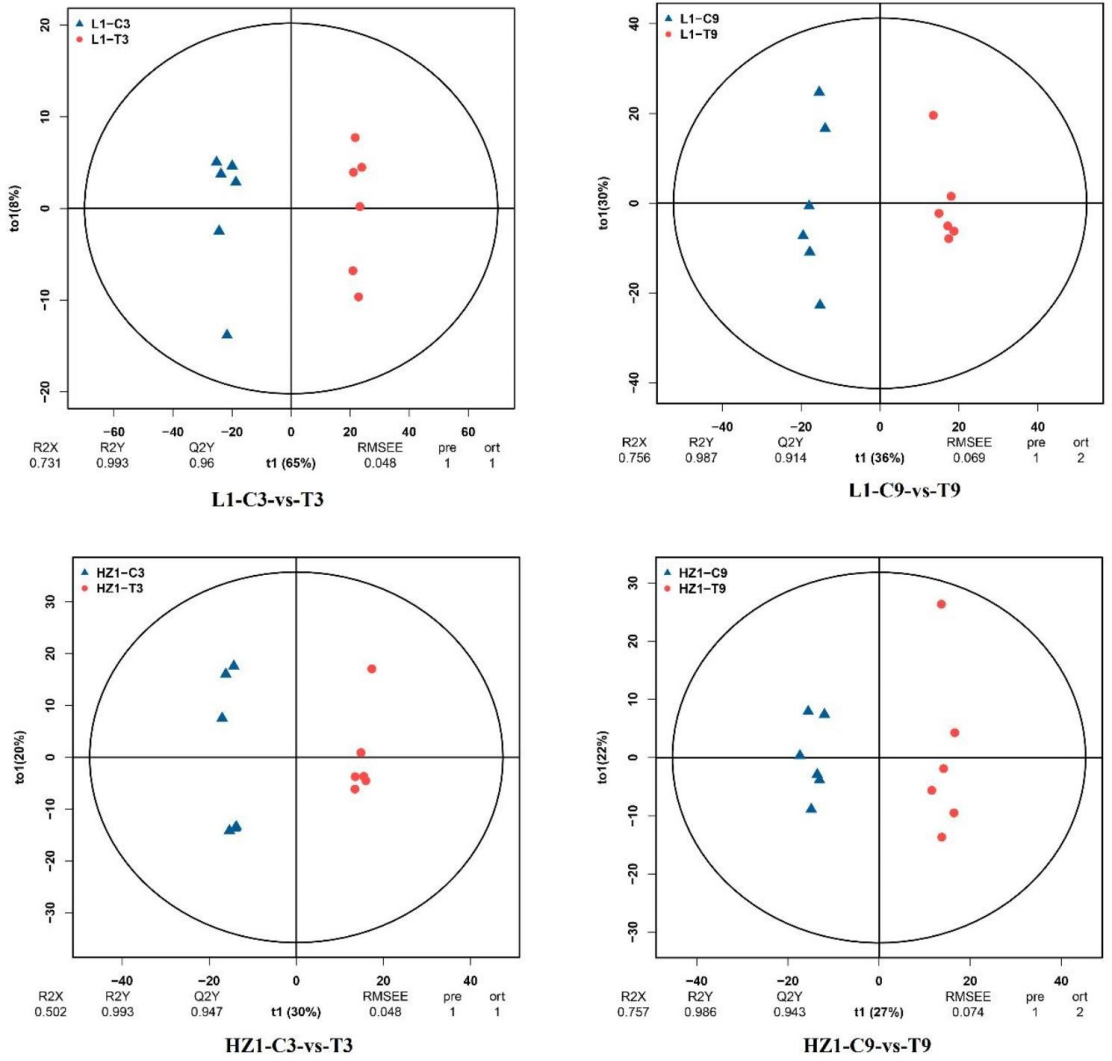
OPLS-DA scatter plot of L1 and HZ1 leaves under drought stress

As shown in Fig. 3, significant segregation was observed in all four treatments under drought stress, with 95% confidence intervals for each group. The cumulative values of R2Y and q 2 in OPLS-DA plots were 0.993 and 0.960(a), respectively, on Day 3 of L 1 drought stress, and 0.993 and 0.960(a), respectively, on Day 9 of L 1 drought stress, the cumulative values of R2Y and Q2 in OPLS-DA plots were 0.987 and 0.914, respectively, and the cumulative values of R2Y and Q2 in OPLS-DA plots were 0.993 and 0.947, respectively, on the third day of HZ1 drought stress The cumulative values of R2Y and Q2 in OPLS-DA plots were 0.986 and 0.943(a), respectively, on the 9th day of HZ1 drought stress. The results show that the OPLS-DA model is not over-fitting and has high reliability and repeatability, which can be used in the follow-up analysis.

### Identification of different metabolites in leaves of different quinoa cultivars under drought stress

Based on the OPLS-DA model, the differential metabolites (DEMS) were screened by VIP value (VIP > 1) and P < 0.05. In L1,523 DEMs were identified on the 3rd day under drought stress, of which 102 DEMs were up-regulated and 421 DEMs were down-regulated, and 406 DEMs were identified on the 9th day under drought stress (Table 3, Table S1-Table S2), of which 140 DEMs were up-regulated, 266 DEMs downgraded. In HZ1,301 DEMs were identified, 177 of which were up-regulated and 124 were down-regulated by drought stress on Day 3, and 272 were identified by drought stress on Day 9, and 136 of which were up-regulated by drought stress on Day 9, 136 DEMs were downgraded. At the same time, it is obvious that the number of up-regulated DEMs in L 1 is always less than the number of down-regulated DEMs.

**Table 3:** basic information of differential metabolites

Group	Total number	Diff number	Up number	Down number
HZ1-C3-vs-T3	846	301	177	124
L1-C3-vs -T3	846	523	102	421
HZ1-C9-vs -T9	846	272	136	136
L1-C9-vs -T9	846	406	140	266

### Analysis of common differential metabolites

**Fig. 4:**
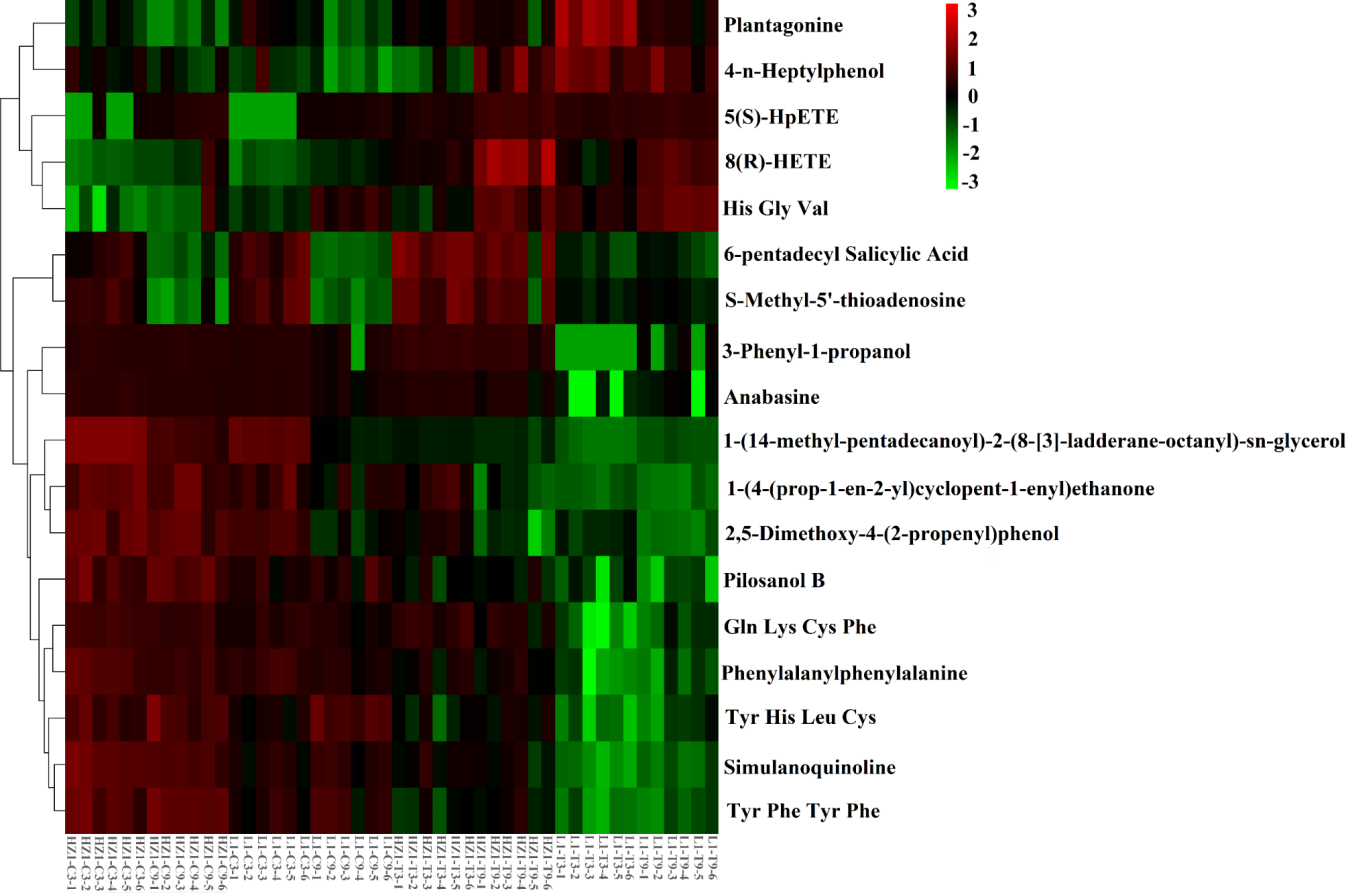
Heatmap of expression of common 18 differential metabolites in four contrast conditions (HZ1-C3-VS-T3, L1-C3-vs-T3, HZ1-C9-vs-T9 and L1-C9-vs-T9).

In 4 group comparisons (HZ1-C3-VS-T3, L1-C3-vs-T3, HZ1-C9-vs-T9 versus L1-C9-VS-T9), we found 59 differentially expressed metabolites that were co-expressed; Among them, 47 differential metabolites were all down-regulated in the comparison of 4 groups after drought stress, and 5 differential metabolites were all up-regulated in the comparison of 4 groups after drought stress, 5(S)-hpete, Theasapogenol a, 8(R)-HETE, planagonine and His Gly Val; 3-phenyl-1-propanol were up-regulated in HZ1 and down-regulated in L1. Among them, 18 metabolites showed significant differences between treatment and control (Fig. 4). Compared with the control, the high accumulation of metabolites in plants under drought conditions included organic acids (5(S)-hpete, 8(R)-HETE and planagonine) and amino acids (His Gly Val). On the other hand, metabolites that show reduced levels under drought include quinoline, borax alcohol B, amino acids (Tyr His Leu Cys, Gln Lys Cys Phe, Tyr Phe Tyr Phe), L-Phenylalanine, 1-(14-methyl-pentadecyl) -2-(8- [3]-gradient alkane-octyl)-tin-glycerol, Chenopodium, 1-(4-phenyl-1-yl) ethyl ](prop-2-en-1-yl) amine, 2, 5-dimethoxy-4-(1-phenylpropyl-2-enyl) phenol. The model of metabolite clustering clearly shows the metabolic changes under different water conditions.

### Unique expression of differential metabolites

**Fig. 5:**
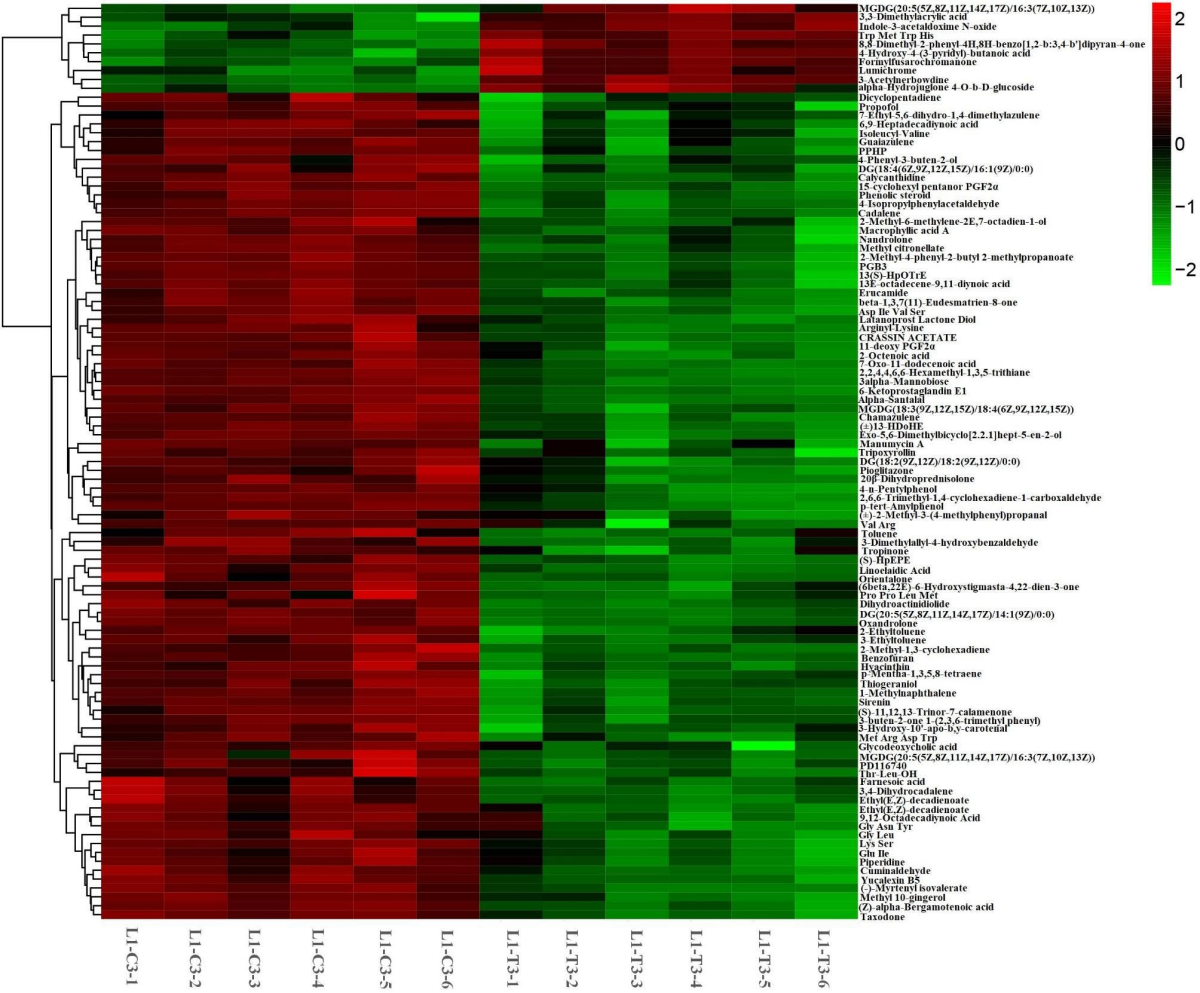
Differentially expressed metabolites in L1 on day 3 of drought stress

At the same time, the differentially expressed metabolites in HZ1 and L1 on the 3rd and 9th day of drought stress were analyzed (Fig. 5). 103 unique differentially expressed metabolites (Table S3) were found in L1 on day 3 of drought stress, of which 93 differentially expressed metabolites were downregulated and 10 differentially expressed metabolites were upregulated. Based on the LOG2FC value, the contents of Erucamide, Lys Ser, 6-Ketoprostaglandin e 1,7-Oxo-11-dodecenoic acid, p-tert-Amylphenol and Glycodeoxycholic acid were significantly down-regulated under drought stress, the contents of 8-Dimethyl-2-phenyl-4H, 8H-benzo [1,2-b: 3,4-b’ ] dipyran-4-one, a kind of flavonoid, 3,3-Dimethylacrylic acid, 4-hydroxy-4-(3-pyridyl)-butanoic acid, 3-Acetylnerbowdine and polypeptide (Trp Met Trp His) were significantly down-regulated under drought stress.

**Fig. 6:**
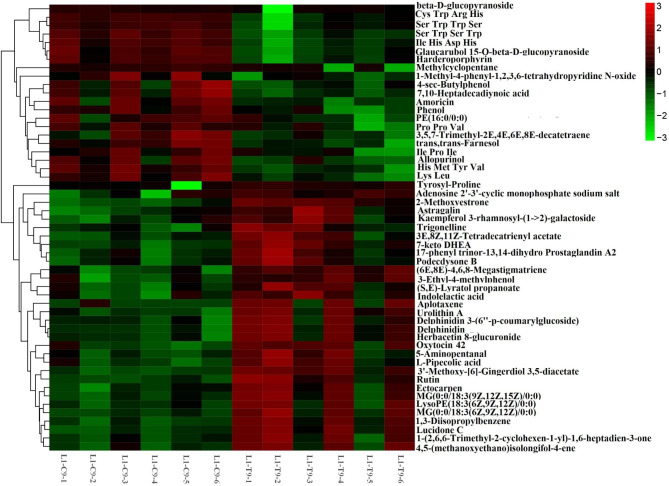
Differentially expressed metabolites in L1 on day 9 of drought stress

There were 53 differentially expressed unique metabolites (Table S3) in L1 on day 9 of drought stress, of which 21 differentially expressed metabolites were downregulated and 32 differentially expressed metabolites were upregulated (Fig. 6). Based on LOG2FC values, the contents of polypeptides (Ile His Asp His, His Met Tyr Val, Cys Trp Arg His), trans, trans-Farnesol, 3,4,7-Trihydroxy-5-methoxy-8-prenylflavan, 4-o-(beta-d-xylopyranosyl-(1-& GT; 6) -beta-d-glucopyranoside) and Methylcyclopentane were significantly decreased under drought stress, the contents of Aplotaxene, Tyrosyl-Proline, 1,3-Diisopropylbenzene, 3’-methoxy- [6]-gingerdiol 3,5-diacetate were significantly up-regulated under drought stress.

**Fig. 7:**
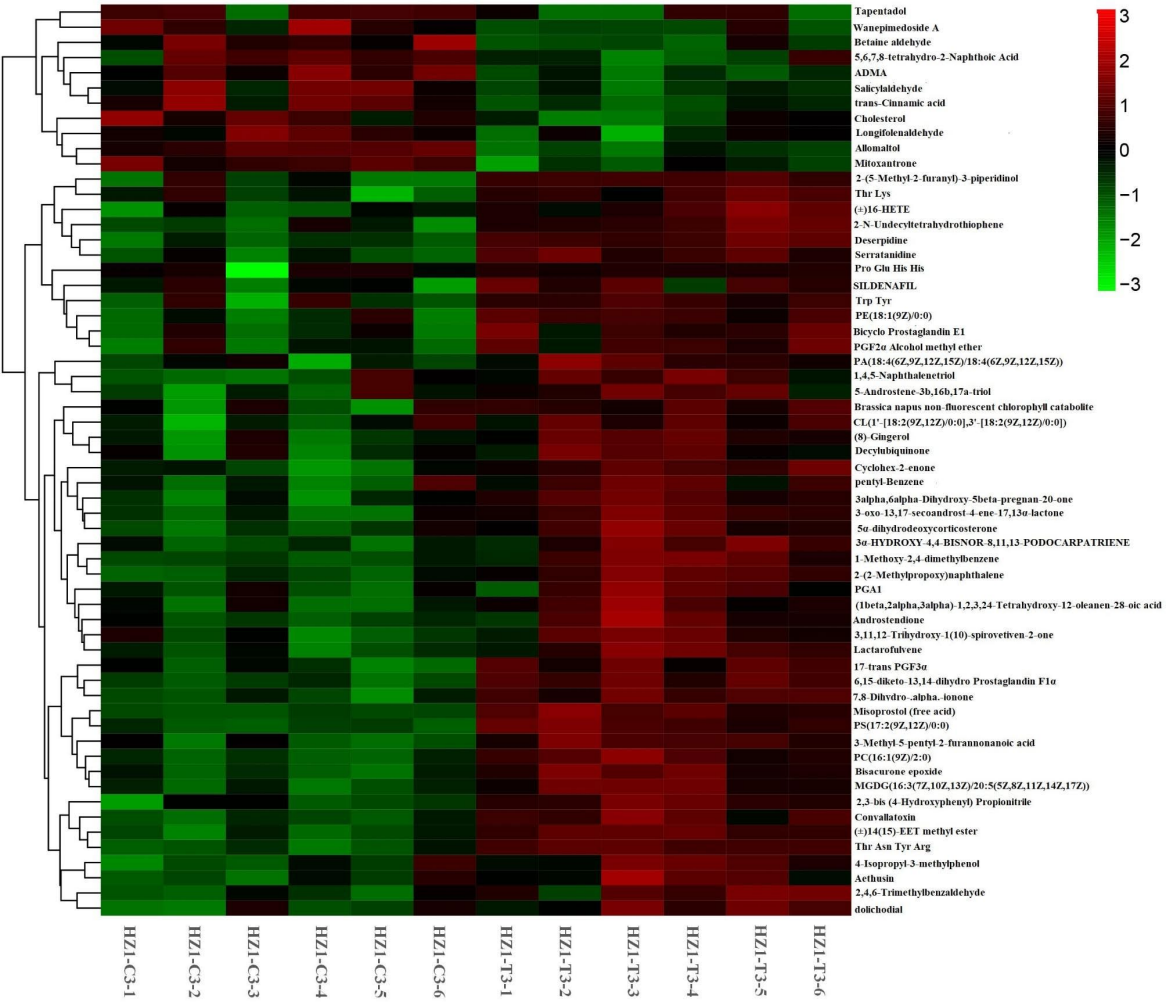
Differentially expressed metabolites of HZ1 on day 3 of drought stress

There were 60 uniquely differentially expressed metabolites (Table S3) in HZ1 on day 3 of drought stress, of which 11 differentially expressed metabolites were downregulated and 49 differentially expressed metabolites were upregulated (Fig. 7). Based on the LOG2FC values, the contents of Tapentadol, 5,6,7,8-tetrahydro-2-Naphthoic Acid and Longifolenaldehyde were significantly decreased under drought stress, the contents of 5-Naphthalenetriol and (2-(5-Methyl-2-furanyl) -3-piperidinol were significantly increased under drought stress.

**Fig. 8:**
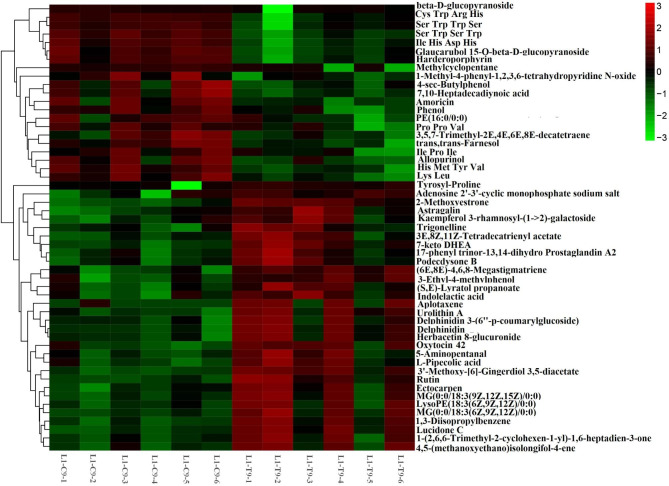
Differentially expressed metabolites of HZ1 on day 9 under drought stress

There were 34 uniquely differentially expressed metabolites (Table S3) in HZ1 on Day 9 under drought stress, of which 13 differentially expressed metabolites were downregulated and 21 differentially expressed metabolites were upregulated (Fig. 8). Based on the LOG2FC value, the contents of 4’-methyl-α-pyrrolidinohexanophenone, Coroglaucigenin-3-o-alpha-l-rhamnopyranoside and Ser Leu Ala were significantly down-regulated under drought stress, while the contents of Sabinol, alpha-Phellandrene dimer and soladulcidine were significantly up-regulated under drought stress.

### Screening of main DEMs

Based on the log2FC value, we screened the DEMS which were different from the control and drought treatments (Table 4). In the comparison of L1-C3-vs-T3, we found that organic acids (5(S)-hpete), volatile compounds (E) -4,8-dimethyl-1,3, the contents of 7-nonyltriene, lovastatin acid, 3-methylene indole and amino acids (glutamic acid, glycine, L-Aspartic Acid and tryptophan) increased in leaves under drought stress. And the contents of Ovalitenin B, Anabsine, p-cresol and 3-phenyl-1-propanol decreased significantly under drought conditions. On the 9th day of drought stress, the contents of Aplotaxene, L-Histidinol, Zidovudine in L1 showed an increasing trend in the leaves under drought stress compared with those in L1-C9-vs-T9. The contents of alkaloids such as pseudoequine, wool phenol (Pubescenol), 3-phenyl-1-propanol, Alkyl cycloalkane (Methylcyclopentane) and phenylalanine decreased significantly under drought conditions.

**Table 4:** Important metabolites with L1-C3-vs -T3 and L1-C3-vs -T3

	L1-C3-vs -T3	Fold_change	log2FC	Pvalue	VIP	regulated
L1-C3-vs -T3	5(S)-HpETE	52.622	5.718	0.001	1.117	up
	(E)-4,8-Dimethyl-1,3,7-nonatriene	43.455	5.441	0.000	1.223	up
	Lovastatin acid (Mevinolinic acid)	13.936	3.801	0.007	1.010	up
	Glu Gly Asp Trp	11.741	3.554	0.004	1.054	up
	3-Methyleneoxindole	10.738	3.425	0.000	1.198	up
	Ovalitenin B	0.016	-5.955	0.000	1.167	down
	Anabasine	0.010	-6.701	0.000	1.220	down
	gamma-L-Glutamyl-gamma-L-glutamyl-L-methionine	0.003	-8.559	0.005	1.077	down
	p-cresol	0.000	-35.542	0.000	1.171	down
	3-Phenyl-1-propanol	0.000	-36.999	0.001	1.170	down
L1-C9-vs -T9	Aplotaxene	5134.867	12.326	0.044	1.110	up
	L-Histidinol	29.459	4.881	0.011	1.349	up
	Zidovudine	28.479	4.832	0.007	1.375	up
	Lovastatin acid (Mevinolinic acid)	10.977	3.456	0.005	1.417	up
	Tricetanidin	10.165	3.346	0.011	1.297	up
	Anabasine	0.157	-2.667	0.013	1.269	down
	Pubescenol	0.121	-3.050	0.007	1.314	down
	3-Phenyl-1-propanol	0.121	-3.052	0.037	1.122	down
	Methylcyclopentane	0.111	-3.170	0.030	1.183	down
	Phenylalanylphenylalanine	0.110	-3.187	0.000	1.540	down

On the 3rd day of drought stress of HZ1 (Table 5), in the comparison of HZ1-c3-vs-T3, we found that the contents of organic acid (5(S)-Hpete), 15(R)-tablet thromboxane A2, one of the Phosphatidylinositol (PI), L-Asparagine-arginine and Peptides (histidine, glycine and leucine) in HZ1 tended to increase under drought stress. And the contents of quercetin 3-β-d-glucoside, phenylalanine, Ovalitenin B, Tapentadol were decreased. On the 9th day of drought stress, we found that the contents of hcl (soladucidine), APIRENE (alpha-Phellandrene dimer), 5-acetyl-3,4-dihydro-2 h-pyrrole, l-histidine (L-Histidinol) and 15(R)-tablet thromboxane a 2 in HZ1 tended to increase under drought stress. However, the contents of fern lactam (Pterolactam), NO inhibitor (V-PYRRO/No), 2,5-dimethoxy-4-(1-phenylpropyl-2-enyl) phenol, pyroglutamic acid and secondary amide (Myrtine) decreased significantly under drought condition.

**Table 5:** Important metabolites with HZ1-C3-vs -T3 and HZ1-C9-vs -T9

	HZ1-C3-vs -T3	Fold_change	log2FC	Pvalue	VIP	regulated
HZ1-C3-vs -T3	15(R)-Pinane Thromboxane A2	69.659	6.122	0.020	1.277	up
	His Gly Val	7.286	2.865	0.044	1.143	up
	5(S)-HpETE	7.249	2.858	0.027	1.274	up
	PI (16:0/22:4(10Z,13Z,16Z,19Z))	6.104	2.610	0.000	1.656	up
	Aspartyl-Arginine	4.972	2.314	0.000	1.689	up
	Quercetin 3-β-D-glucoside	0.283	-1.823	0.001	1.539	down
	Phenylalanylphenylalanine	0.257	-1.960	0.002	1.554	down
	Ovalitenin B	0.091	-3.465	0.000	1.743	down
	Tapentadol	0.055	-4.188	0.021	1.313	down
	1-(14-methyl-pentadecanoyl)-2-(8- [3]-ladderane-octanyl)-sn-glycerol	0.051	-4.306	0.000	1.820	down
HZ1-C9-vs -T9	soladulcidine	333.724	8.383	0.003	1.644	up
	alpha-Phellandrene dimer	19.837	4.310	0.046	1.242	up
	5-Acetyl-3,4-dihydro-2 H-pyrrole	13.238	3.727	0.003	1.671	up
	L-Histidinol	12.244	3.614	0.032	1.332	up
	15(R)-Pinane Thromboxane A2	10.688	3.418	0.045	1.200	up
	Pterolactam	0.233	-2.105	0.020	1.396	down
	V-PYRRO/NO	0.216	-2.210	0.018	1.423	down
	1-(14-methyl-pentadecanoyl)-2-(8- [3]-ladderane-octanyl)-sn-glycerol	0.125	-2.998	0.000	1.817	down
	Pyroglutamic acid	0.107	-3.218	0.007	1.532	down
	Myrtine	0.087	-3.529	0.002	1.718	down

### KEGG metabolic analysis of differential metabolites

**Fig. 9:**
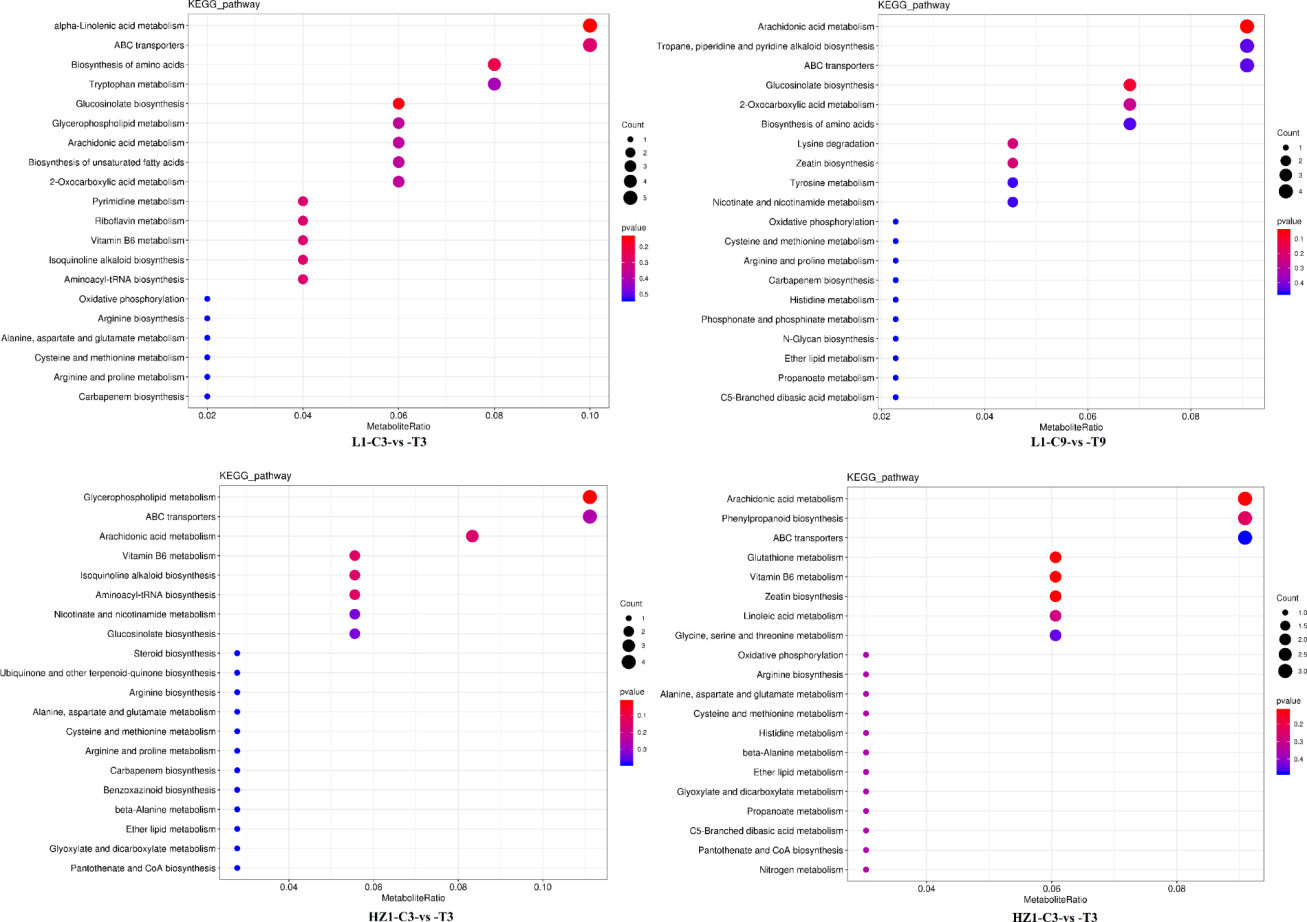
Bubble plots of KEGG enrichment pathway under four contrast conditions of top 20 (HZ1-C3-VS-T3, L1-C3-vs-T3, HZ1-C9-vs-T9 and L1-C9-vs-T9). Note: We have obtained the rights holder’s copyright permission from KEGG officials, who allowed us to modify the KEGG images for this study as appropriate.

In order to further identify the key metabolic pathways of two quinoa materials under drought stress, KEGG enrichment analysis of identified DEMS was carried out. Further analysis showed that 43(50 DEMs) and 42(44 DEMs) metabolic pathways were enriched in L 1 on Days 3 and 9 under drought stress, respectively, the top 20 metabolic pathways were showed in Fig. 9. On Day 3 of drought stress, 50 DEMs in L 1 were assigned to 43 metabolic pathways, with alpha-Linolenic acid metabolism (KO00592) being associated with ABC transporters; (KO02010) pathway having the most DEMs (5,10%) ; followed by Biosynthesis of amino acids (KO01230) pathway (4,8%), Tropane, piperidine and pyridine alkaloid biosynthesis (KO00960) pathway (4,8%), Tryptophan metabolism (KO00380) pathway (4,8%), Glucosinolate biosynthesis (KO00966) pathway (3,6%), Glycerophospholipid metabolism; KO00564 pathway (3,6%), Arachidonic acid metabolism (KO00590) pathway (3,6%), Biosynthesis of unsaturated fatty acids (KO01040) pathway (3,6%) and 2-Oxocarboxylic acid metabolism (KO01210) pathway (3,6%). In addition, 10 metabolic pathways contained 2 DEMs and 23 metabolic pathways contained 1 DEMs respectively. Meanwhile, alpha-Linolenic acid metabolism (KO00592) and Glucosinolate biosynthesis (KO00966) pathways were significantly enriched. On the 9th day of drought stress, 44 DEMs were distributed to 42 metabolic pathways in L 1, and the Arachidonic acid metabolism (KO00590), Tropane, piperidine and pyridine alkaloid biosynthesis (KO00960) and ABC transporters (KO02010) pathways had the most DEMs (4,9.09%) ; Secondly, Glucosinolate Biosynthesis (KO00966) pathway (3,6.82%), 2-Oxocarboxylic acid metabolism (KO01210) pathway (3,6.82%), Biosynthesis of amino acids (KO01230) pathway (3,6.82%), Tryptophan metabolism (KO00380) pathway (3,6.82%), alpha-Linolenic acid metabolism (KO00592) pathway (3,6.82%), in addition, 5 metabolic pathways contained 2 DEMS and 29 metabolic pathways contained 1 DEMs. Meanwhile, Arachidonic acid metabolism (KO00590) and Glucosinolate biosynthesis (KO00966) pathways were significantly enriched. 42(37 DEMs) and 37(34 DEMS) metabolic pathways were enriched in HZ1 on Day 3 and Day 9 under drought stress, respectively.

On Day 3 of drought stress, 37 DEMs in HZ1 were assigned to 42 metabolic pathways, with the most DEMs (4,11.11%) in the Glycerophospholipid metabolism (KO00564) versus ABC transporters (KO02010) pathway; The next were Arachidonic acid metabolism (KO00590) pathway (3,8.33%), Tryptophan metabolism (KO00380) pathway (3,8.33%), Vitamin B 6metabolism (KO00750) pathway (5.5.56%), Isoquinoline alkaloid biosynthesis (KO00950) pathway (5.5.56%), Aminoacyl-tRNA biosynthesis (KO00970) pathway (5.5.56%), Nicotinate and nicotinamide metabolism (KO00760) pathway (5.5.56%), Glucosinolate biosynthesis (KO00966) pathway (5.5.56%), Glycine, Glycine, Glycine, Glycine and nicotinamide, serine and threonine metabolism (Ko00260) pathway (5.5.56%), 2-Oxocarboxylic acid metabolism (KO01210) pathway (5.5.56%), Phenylpropanoid Biosynthesis (KO00940) pathway (5.5.56%), Biosynthesis of amino acids (KO01230) pathway (5.5.56%), alpha-Linolenic acid metabolism (KO00592) pathway (5.5.56%) and Tropane, piperidine and pyridine alkaloid biosynthesis (KO00960) pathway (5.5.56%). In addition, 27 metabolic pathways each contained 1 DEMs, and the significantly enriched metabolic pathway was Glycerophospholipid metabolism (KO00564). On the 9th day of drought stress, 34 DEMs in HZ1 were assigned to 37 metabolic pathways. Arachidonic acid metabolism (KO00590) pathway (3,9.09%), Phenylpropanoid biosynthesis (KO00940) pathway (3,9.09%), and ABC transporters (KO02010) pathway had the most DEMs (3,9.09%) Glucosinolate Biosynthesis (KO00966) pathway (2,6.06%), Vitamin B 6metabolism (KO00750) pathway (2,6.06%), Zeatin Biosynthesis (KO00908) pathway (2,6.06%), Linoleic acid metabolism (KO00591) pathway (2,6.06%), Glycine, serine and threonine metabolism (Ko00260) pathway (2,6.06%), Biosynthesis of amino acids (KO01230) pathway (2,6.06%), Tropane, piperidine and pyridine alkaloid biosynthesis (KO00960) pathway (2,6.06%). In addition, there were 27 metabolic pathways with 1 dems, and the Vitamin B 6 metabolism (KO00750) pathway and Zeatin biosynthesis (KO00908) pathway were significantly enriched.

### α-Linolenic acid metabolism pathway analysis

**Fig. 10:**
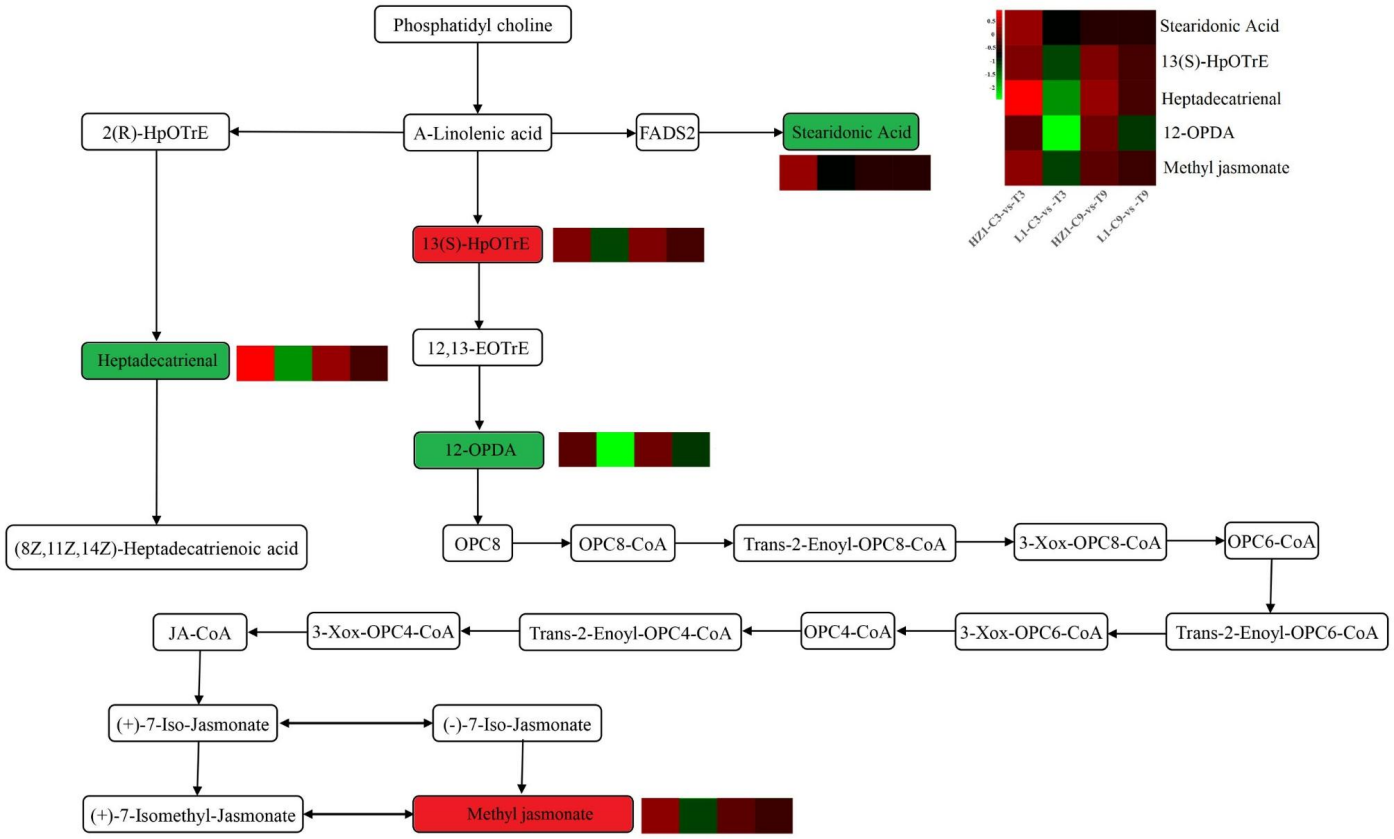
The α-Linolenic acid metabolism pathway based on the KEGG pathway

Based on the KEGG metabolic pathway, we found that the α-Linolenic acid metabolism pathway was enriched in many different metabolites (Fig. 10). We found that the contents of Stearidonic Acid, 13(s)-HPOTRE, Heptadecatrienal, 12-OPDA and Methyl jasmonate were significantly down-regulated under L1-C3-VS-T3 treatment, and the contents of Stearidonic Acid, 12-OPDA and Methyl jasmonate were also significantly down-regulated under L1-C9-VS-T9 treatment. However, the contents of Heptadecatrienal and Methyl jasmonate were significantly up-regulated under HZ1-C3-VS-T3 treatment. Interestingly, these differential metabolites were found to be down-regulated in the drought-resistant genotype L1 and up-regulated in the drought-sensitive genotype HZ1, these results suggested that this change of different metabolites may be different strategies for quinoa materials to cope with drought stress.

## Discussion

The ability of plants to maintain high water status under drought stress is an important strategy for them to cope with drought stress, and changes in relative water content are the best method for assessing water scarcity and physiological water status under water stress [33], higher relative water content is regarded as an indicator of drought tolerance. It was found that RWC in barley decreased with the increase of water stress [34]. Our results showed that the decrease of RWC of L 1 was significantly lower than that of HZ1 under drought stress, and the RWC of L 1 was always higher than that of HZ1. At the same time, the root activity of L1 was always higher than that of HZ1 under drought stress. Therefore, we speculate that this difference in relative water content may be related to the ability of different quinoa materials to absorb water from soil, which also indicates that under drought stress, the sensitive material of quinoa was more susceptible to the decline of relative water content than the drought-tolerant material. These results suggest that different quinoa materials have different sensitivity to mannitol-induced water stress. The enhanced water retention observed in material L1 may play an important role in plant survival under water-deficient conditions, even when challenged by drought stress. Osmoregulation is an important mechanism by which plants maintain water status under drought conditions, in which the accumulation of proline, soluble sugar, soluble protein and betaine is associated with drought tolerance [35]. Studies have shown that plants increase the concentration of cell fluid and decrease its osmotic potential by changing the contents of soluble sugar and soluble protein, which further enhances the ability of plants to absorb water, and ensure cell growth and metabolism [36]. The increase of soluble sugar content can increase the protoplast viscosity, elasticity and cell fluid concentration, and thus improve the water absorption and water retention capacity of crops [37]. Our results showed that the contents of soluble sugar and soluble protein in L1 were higher than those in HZ1 at different time points after drought stress, it is also indicated that L1 materials accumulate more soluble sugar and soluble protein, which leads to better osmotic regulation and stronger protection of photosynthetic organs. Proline plays an important role in the regulation of osmotic pressure, which enables cells to retain more water. Furthermore, it can act as a ROS scavenger and a regulator of cellular redox STATUS30, and proline accumulation is positively correlated with plant tolerance to various environmental stresses. Some studies have found that the proline content of drought tolerant varieties is higher than that of sensitive varieties [38]. In this study, the content of proline in the leaves of PEG-stressed quinoa was significantly higher than that of the control, and the content of proline accumulated in the materials of L1 and HZ1 during 3–9 days after drought stress, the content of proline decreased gradually in L1 and HZ1 in 9–12 days, and the higher proline content in L1 may be caused by the change of expression of drought-responsive genes, this may improve the hydration of plants. The results also strengthened the close relationship between the increase of proline concentration and the relative water content of plants in the drought-resistant mechanism. It was found that the ratio of palisade tissue to spongy tissue increased and leaf density increased with the severity of water deficit, which indicated that the drought resistance of plants was increasing, the relative reduction of the thickness of the developed palisade and spongy tissues helps to improve the water-holding capacity of plants, which indicates that the internal structure of leaves of HZ1 is prone to change significantly after drought stress, the effect of drought stress was much greater than that of L1. In this study, we used metabolomic analysis to dissect metabolite changes in quinoa in response to drought levels during drought. Compared with the control, the high accumulation of organic acids including 5(S)-hpete, 8(R)-HETE, Plantagonine and three amino acids (His, Gly and Val) were observed in plants grown under drought conditions, indicates that they are biomarkers of drought. Organic acids, such as lactic acid, malic acid and succinic acid, are increased in response to drought stress. Although the role of organic acids in drought response and adaptation is not fully understood, they may accumulate as a result of drought-induced tricarboxylic acid cycle perturbations [39, 40].

### Amino acids and their derivatives

The accumulation of amino acids in plants under drought stress, such as aromatic amino acid, is beneficial for plants to cope with drought stress. Increases in amino acid levels are thought to enhance plant resilience by influencing various physiological mechanisms, such as the regulation of osmolarity changes, ROS detoxification, and the regulation of intracellular pH levels [41]. Drought stress conditions enhanced the accumulation of these metabolites regardless of the variety (tolerant or sensitive), but their relative intensity was higher in tolerant varieties. Under drought stress, proline plays a key role as a reactive oxygen species scavenger, and high accumulation of proline may act as an energy source for the maintenance of photosynthetic and respiratory processes [42]. In this study, we found that the content of proline (Table S2) in HZ1 decreased significantly on the 3rd day after drought stress, while the accumulation of proline in L1 increased significantly on the 3rd and 9th day after drought stress. In this study, both HZ1 and L1 showed impairment of photosynthetic capacity; however, the impairment of L1 was significantly lower compared with HZ1, possibly due to the strong accumulation of proline, this helps to protect the photosystem and maintain the redox balance in the photosynthetic membrane. Proline accumulation was previously found to be caused by increased glutamate-mediated biosynthesis, 36 and we found a decrease in some of the transporters of amino acid-related metabolites (L-Glutamine), with both HZ1 and L1 having reduced L-Glutamine content; This reduction is consistent with a shift in metabolic activity toward proline biosynthesis. In addition, the reduction in L-l-glutamine complements the need for amino acid biosynthesis in HZ1 and L1, which is more pronounced in Hz. At the same time, more metabolites were accumulated in L1 than in HZ1. In addition, Valine, L-Isoleucine, threonine and leucine derivatives undergo significant changes during drought stress, resulting in degradation of valine, L-Isoleucine, threonine and leucine, these amino acids are glycogen amino acids associated with pyruvate metabolism, and increases in these amino acids may be associated with inhibition of protein biosynthesis or enhanced protein degradation [43]. Studies have found that Branched-chain amino acid levels increase in wheat and Arabidopsis under drought conditions and are also regulated at the transcriptional level in Arabidopsis [44].

In this study, we observed that the contents of tyrosine, tryptophan and L-Phenylalanine aromatic amino acid increased under drought stress (Table S2), and a similar result was observed in soybean [45]. The aromatic amino acid accumulated during drought may be used as an alternative energy source to provide quinoa with drought tolerance. As precursors of different secondary metabolites in Shikimic acid pathway, including indoles acetate, lipid precursors, and lignin, aromatic amino acid play a critical role in stress tolerance [46, 47]. The higher tryptophan accumulation in this study contributes to the ROS scavenging mechanism, as evidenced by the higher ROS scavenging enzyme activity under drought stress compared with controls, thus ultimately reducing damage to the quinoa photosystem. At the same time, we also observed an increase in histidine content under drought stress, and studies have found that histidine may be involved in cadmium resistance and accumulation by reducing oxidative damage [47, 48]. Previous studies have found that tyrosine content is closely related to plant drought resistance, and that L-Phenylalanine content in HZ1 decreased significantly, this is consistent with studies in chickpeas, 15 suggesting that L-Phenylalanine and tyrosine are important traits associated with drought resistance.

#### Nucleotides and their derivatives

Nucleotide metabolism is one of the important components of metabolic pathways, in which nucleotide is an important component of nucleic acid. For the synthesis of carbohydrates, lipids, peptides, and secondary metabolites, nucleic acids provide nucleic acids as the ultimate energy source [49) were identified in HZ1 and L1, respectively. In HZ1,3’-o-methylguanosine, Succinoadenosine, S-Methyl-5’-thioadenosine, 2-Thiocytidine, Purine, Azacitidine, Isopentenyl adenosine and Zidovudine changed in different degrees Succinoadenosine, S-Methyl-5’-thioadenosine, Cytosine, D-Guanosine, Azacitidine, 2-Aminomethylpyrimidine, cGMP, 2-Thiocytidine, Isopentenyl adenosine in L 1 significantly changed, suggesting that Quinoa protects nucleic acids by altering purine and pyrimidine metabolism in leaves under drought stress, however, purine and pyrimidine metabolism in L 1 leaves were susceptible to drought stress.

#### Lipid metabolites

Plant lipids are diverse and essential to cells. As hydrophobic barriers of membrane, they are essential for the integrity of cells and organelles. Furthermore, lipids are stored in seeds in the form of chemical energy, and they act as signaling molecules to regulate cellular metabolism [50]. The major form of lipids in plants is glycerol, and lipid synthesis involves several organelles in the cell. Fatty acids are synthesized by chloroplasts and are the major components of the chloroplast membrane. Fatty acids are transferred to the cytoplasm and bound to glycerol in the endoplasmic reticulum to form a phospholipid. In the endoplasmic reticulum of epidermal cells, fatty acids are converted into components of the cuticle and wax, which are stratum corneum lipids that prevent water loss [51]. In addition, galactolipids play a crucial role in chlorophyll biosynthesis and the accumulation of light-trapping proteins. Plants maintain their integrity and fluidity by altering the lipid composition of cell membranes during dehydration [52]. Therefore, lipid metabolism also plays a key role in drought stress. In addition, the increase in unsaturated fat levels was related to the level of drought resistance in plants. The higher the level of accumulation of unsaturated fat lipids, the stronger the drought tolerance of plants, drought-tolerant plants increased unsaturated fat levels during drought [53]. In this study, we found that different expression unsaturated fat were found in L1 and HZ1 under drought stress (Table S2), 5(s)-hydropericosapentaenoic acid (5(S)-hpete) and 8(R)-hydroxy-(5Z, 9E, 11Z, 14Z)-Eicosatetraenoic acid (8(R)-HETE) showed an increasing trend in both materials, the content of 7-Tridecynoic acid in L1 increased under drought stress and decreased in HZ1, indicating that drought stress significantly reduced the content of unsaturated fat in HZ1 leaves, further affect their drought resistance. The disorder of plant physiological function is related to the abnormality of biofilm composition under stress. Membrane integrity plays an important role in plant drought resistance [54]. Previous studies [55] found that there were significant differences in lipid, phospholipid and fatty acid contents between two Selaginella tamariscina cultivars under drought stress. The integrity of lipid bilayer in plasma membrane is affected by the proportion of bilayer phosphatidylcholine (PC) and non-bilayer phosphatidylethanolamine (PE). PC can be used as a precursor for the synthesis of other glycerides, while PE plays an important role in stress signaling pathways [56]. Our study found that the contents of phosphatidylcholine (PC(16:1(9E)/0:0) and PC (14:0/O-1:0)) and non-bilayer phospholipid ethanolamine PE (17:0/0:0) in L1 and HZ1 increased significantly on the 3rd day of drought stress, the content of these three metabolites in HZ1 was higher than that in L1, and PC could also induce cell necrosis. In this study, the content of lysophosphatidylcholine in HZ1 was higher than that in L1, it is suggested that HZ1 produces more LPC, which leads to more serious damage to its cell membrane, and further indicates that the cell membrane of HZ1 suffers more damage under drought stress.

## Conclusion

The results showed that the leaves treated with 20% PEG6000 in quinoa genotypes with different sensitivity to drought had different mechanisms of metabolite accumulation and regulation, it is of great significance to better understand the mechanisms of abiotic stress tolerance. In a word, polypeptides, organic acids and amino acids are the main metabolites changed by drought stress. Tyr His Leu Cys, Gln Lys Cys Phe, Tyr Phe Tyr Phe, pentadecanoic acid, 5(S)-hpete, 8(R)-HETE, proline, L-Phenylalanine were shown after 20% PEG 6000 treatment, they can be considered as major drought stress-specific markers and osmoprotectants. Metabolomics approaches will improve our understanding of the mechanisms underlying quinoa dehydration and will become increasingly important in the future.

### Electronic supplementary material

Below is the link to the electronic supplementary material.

Supplementary Material 1

Supplementary Material 2

Supplementary Material 3

Supplementary Material 4

Supplementary Material 5

Supplementary Material 6

## Data Availability

The datasets analysed during this study are included in this published article and its supplementary information files.
