# Supplementary figures and images for: Non-targeted metabolomics analysis of metabolite changes in two quinoa genotypes under drought stress
Source: BMC Plant Biol. 2023 Oct 20;23:503. doi: 10.1186/s12870-023-04467-6 (PMC10588040; doi:10.1186/s12870-023-04467-6)

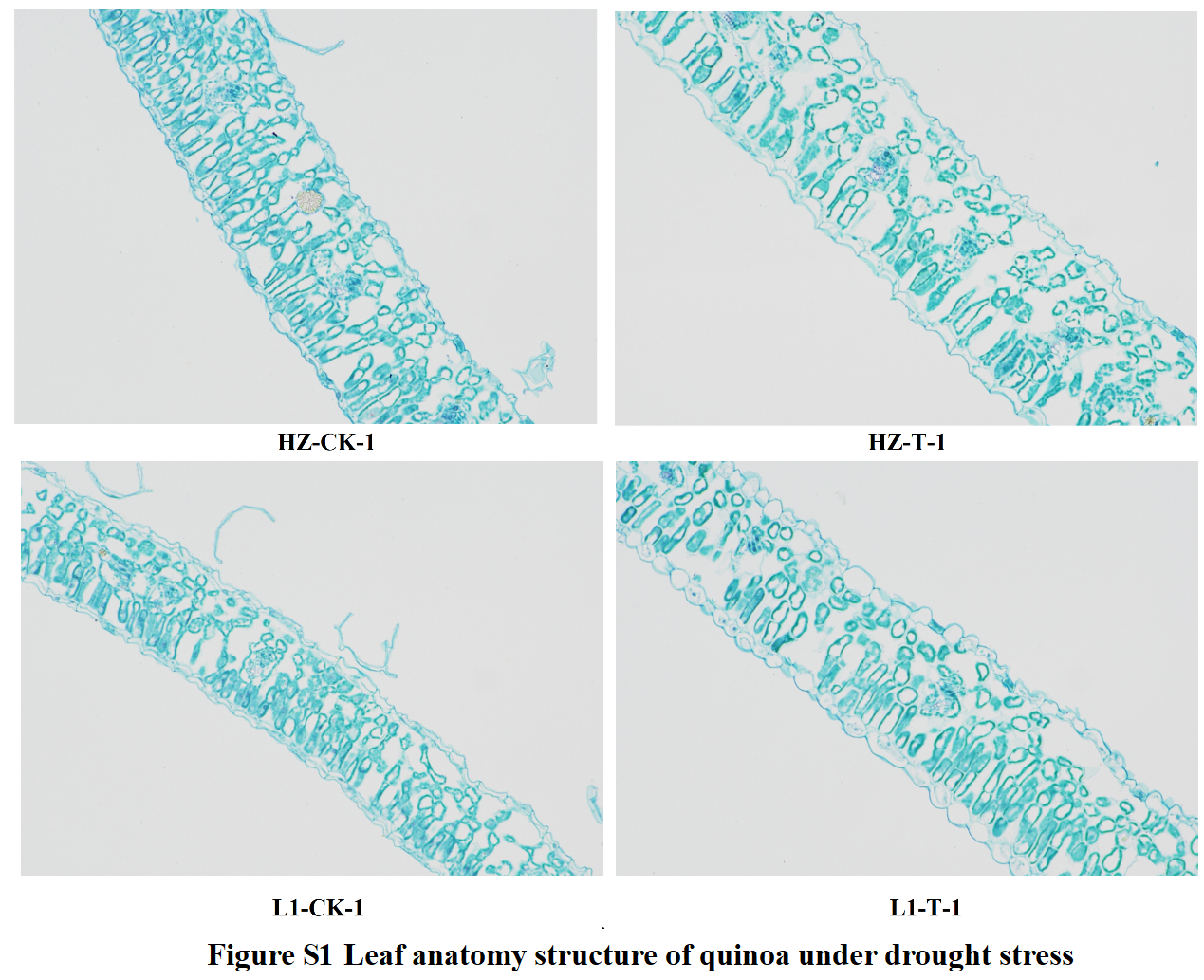

Supplement: Supplementary file 1 — Supplementary Material 1 [file 12870_2023_4467_MOESM1_ESM.tif]

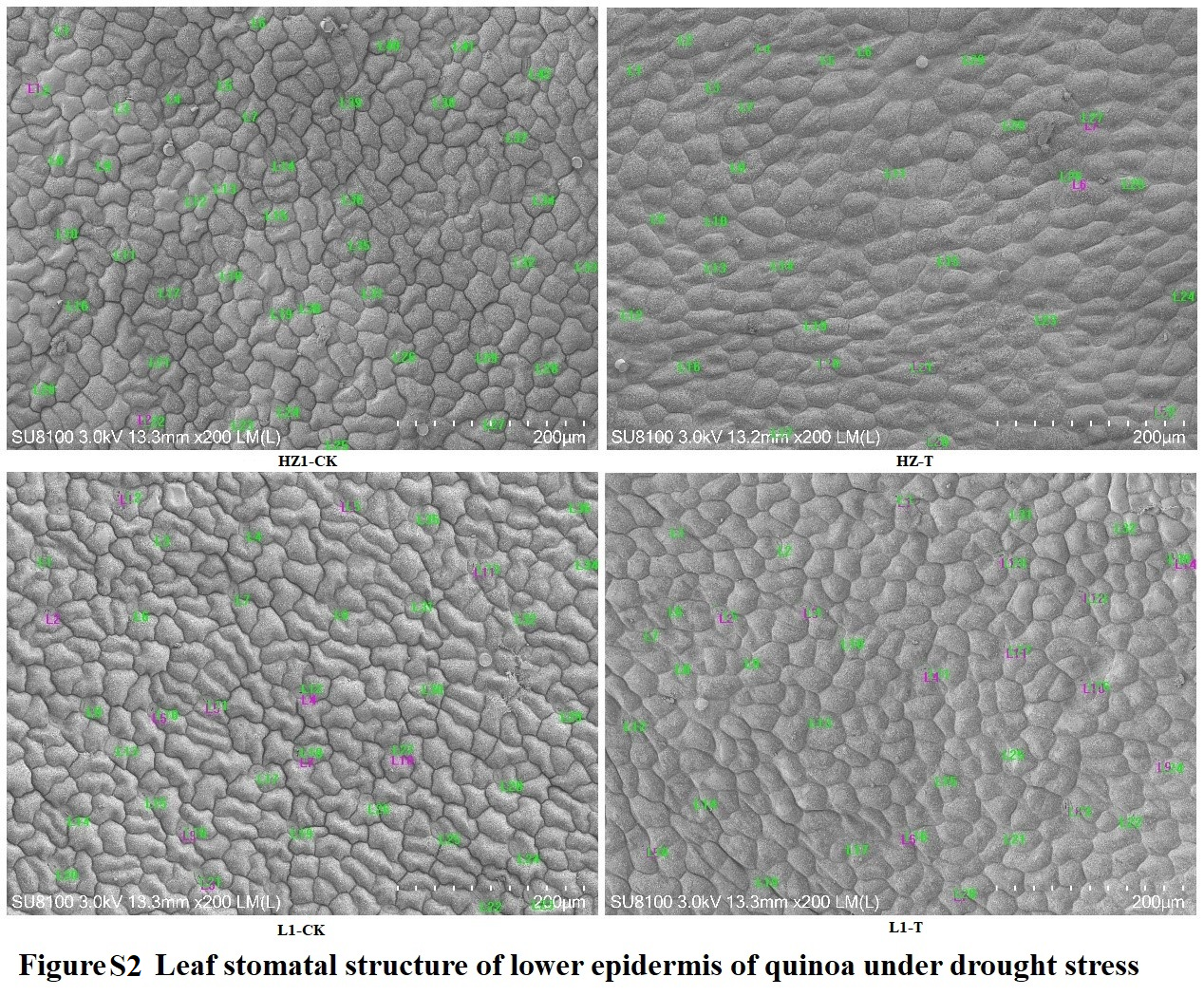

Supplement: Supplementary file 2 — Supplementary Material 2 [file 12870_2023_4467_MOESM2_ESM.tif]

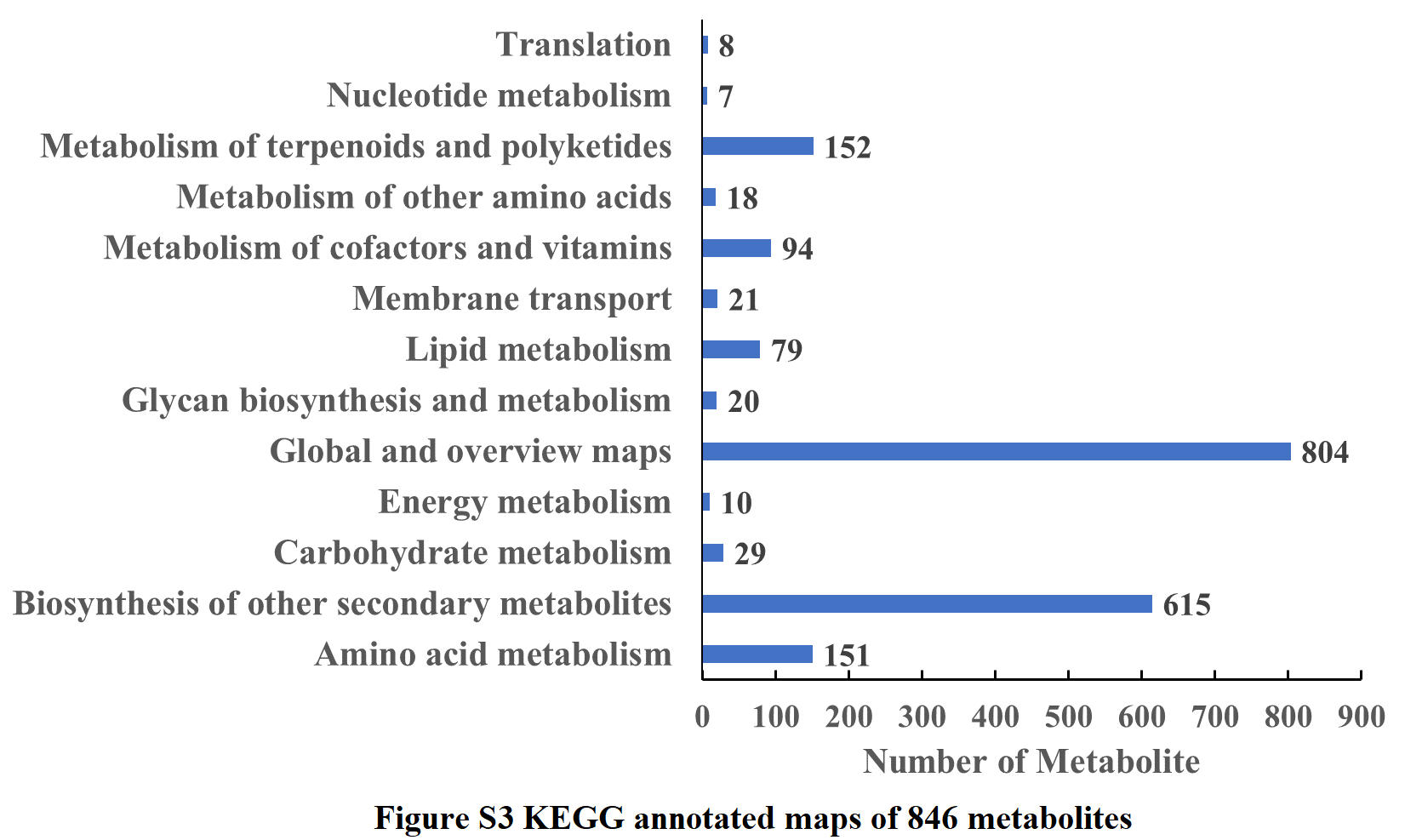

Supplement: Supplementary file 3 — Supplementary Material 3 [file 12870_2023_4467_MOESM3_ESM.tif]
